# Glossary

**Published:** 1996

Agoraphobia: A mental disorder characterized by an irrational fear of leaving the familiar setting of home or venturing into the open. This disorder is so pervasive that a large number of external life situations are entered into reluctantly or avoided; often associated with *panic attacks*.
Alcohol-induced disorders: A group of psychiatric symptoms that develop in the course of intoxication or withdrawal from alcohol. These symptoms, including depression and anxiety, tend to be temporary and disappear within days or weeks of abstinence. Because they are short lived, the symptoms rarely require long-term counseling or psychotherapy, and little evidence indicates that they benefit from medications.
Antisocial personality disorder: A pervasive pattern of disregard for and violation of the rights of others. The disorder is characterized in part by risk taking, criminality, and pathological lying.
Anxiety disorders: A group of long-term, often lifelong, psychiatric disorders, the central feature of which is some form of nervousness or anxiety. These disorders include *panic disorder, social phobia*, and other long-term anxiety conditions. Often they require active treatment that can involve education and counseling, behavioral treatments (e.g., teaching relaxation techniques or ways of approaching feared situations), and, occasionally, long-term medications.
Anxiety symptoms: Signs include feelings of nervousness, tension, or anxiety. They can include temporary symptoms that involve *panic attacks*, fears, or other nervous behaviors. Sometimes these symptoms occur as a reaction to a life situation; at other times, they are related to the effects of alcohol or other drugs. The symptoms may represent long-term psychiatric disorders that are outside the context of intoxication or stress.
Depressive disorders: Psychiatric conditions characterized by intense sadness and symptoms that include interference with sleep, appetite, and daily life functioning. Unless treated, such symptoms are likely to remain for many months and even years.
Depressive symptoms: General and frequently observed feelings of sadness apparent during periods of grief, reactions to difficult situations, or as consequences of medical conditions or side effects of medications or drugs. The sadness resembles the symptoms seen in *depressive disorders* but usually is short lived and disappears with time.
Dysthymia: A disorder characterized by at least 2 years of depressed mood.
Major depressive episode: A period of at least 2 weeks during which there is either depressed mood or the loss of interest or pleasure in nearly all activities.
Mania: An episode of intense hyperactivity, rapid thoughts, and poor judgment that is likely to last weeks to months unless actively treated. A person who experiences an independent major manic episode usually is diagnosed as demonstrating bipolar, or *manic-depressive*, disorder.
Manic-depressive disorder: A psychiatric disorder characterized by episodes of both manic and *depressive disorders*. This condition also is referred to as bipolar disorder.
Obsessive-compulsive disorder: An *anxiety disorder* characterized by a persistent intrusion of unwanted and uncontrollable thoughts, urges, or actions, such as repetitive hand-washing.
Panic attacks: The abrupt onset of an episode (usually lasting about 10 to 20 minutes) of feelings of intense anxiety or nervousness accompanied by rapid heartbeats (i.e., palpitations) and a feeling of shortness of breath. Panic attacks can occur as a reaction to severe stress, as a complication of some medications (e.g., diet pills), during intoxication with stimulant drugs (e.g., amphetamines or cocaine), or during withdrawal from depressant drugs (e.g., alcohol).
Panic disorder: A long-term, frequently lifelong, *anxiety disorder* characterized by *panic attacks* that occur frequently (i.e., multiple times per month) and develop unrelated to stress, side effects of medications, or effects of alcohol or other drugs. This disorder often responds to education and some behavioral treatments, including relaxation, but may require medications (e.g., antidepressants) in certain cases.
Schizophrenia: A chronic disorder characterized by delusions, hallucinations, and disorganized thoughts and behavior.
Social phobia: A long-term, often lifelong, major *anxiety disorder* characterized by a fear (i.e., a phobia) and avoidance of specific social situations even though he or she knows that the fear is irrational. The avoidance results in repetitive major life problems.

